# Supplementary material for: Quantifying the role of hyaluronic acid on collagen fiber mechanics via femtosecond laser ablation
Source: bioRxiv. 2026 Oct 29:2026.10.27.762233. Preprint. [Version 1] doi: 10.64898/2026.10.27.762233 (PMC13734012; doi:10.64898/2026.10.27.762233)
Supplement: Supplementary file 1 [file media-1.pdf]

Supporting Information

Quantifying the role of hyaluronic acid on collagen fiber mechanics via femtosecond laser ablation

*Hannah M. Larson, Faith Miller, Natalie Hawley, Olivia Ward, Alysse DeFoe, Jason A. Burdick, Sarah Calve**

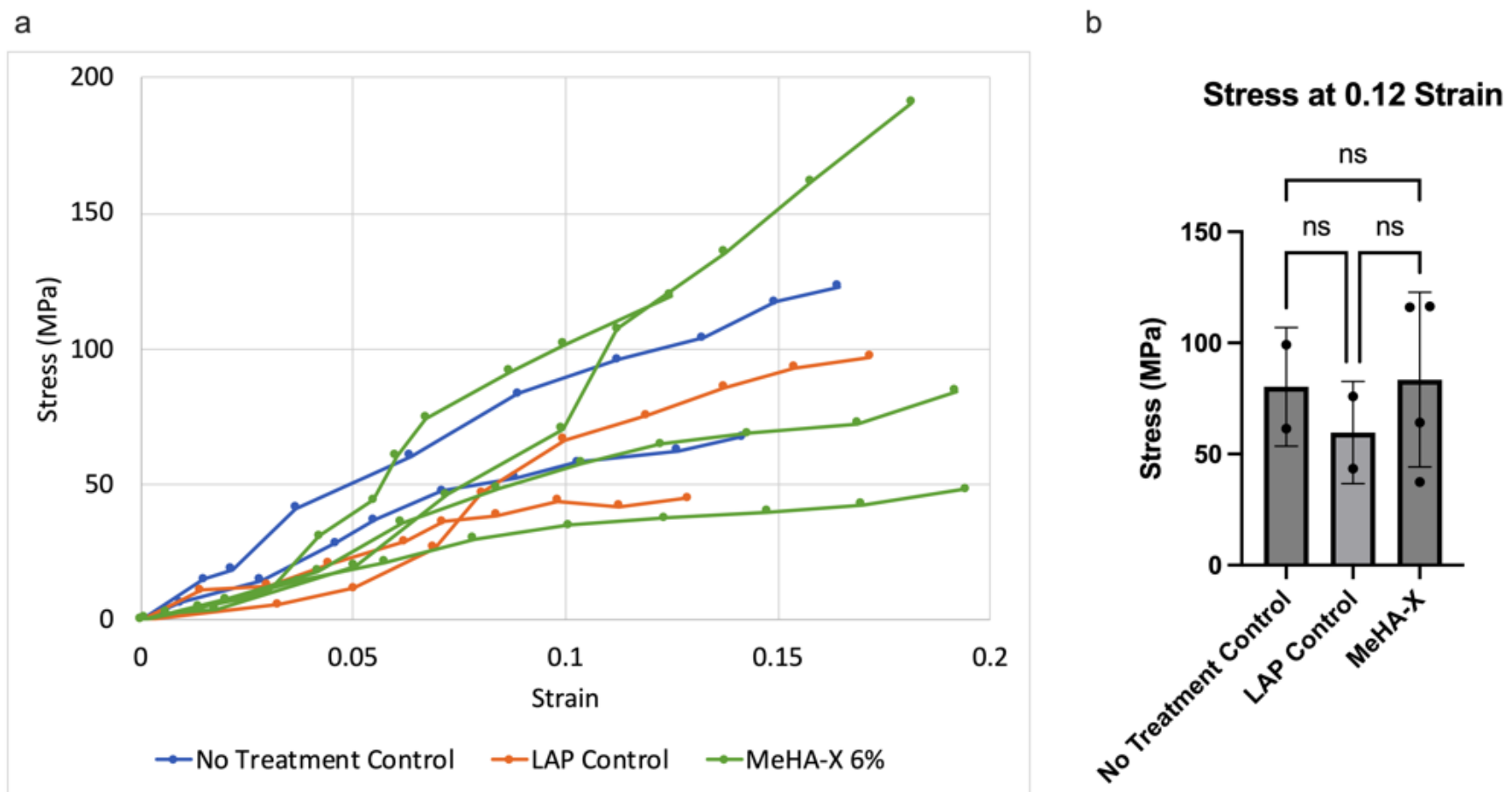

Figure S1. MeHA used in study (6% methacrylation) did not appear to affect tendon macroscale stiffness. a) Stress–strain curves for no treatment control, LAP control, and MeHA-X treated tendons. b) Stress at 0.12 strain. Data represent mean ± SD. Brown–Forsythe one-way ANOVA with Dunnett's T3 post hoc test, n = 2-4 per group, ns = not significant.

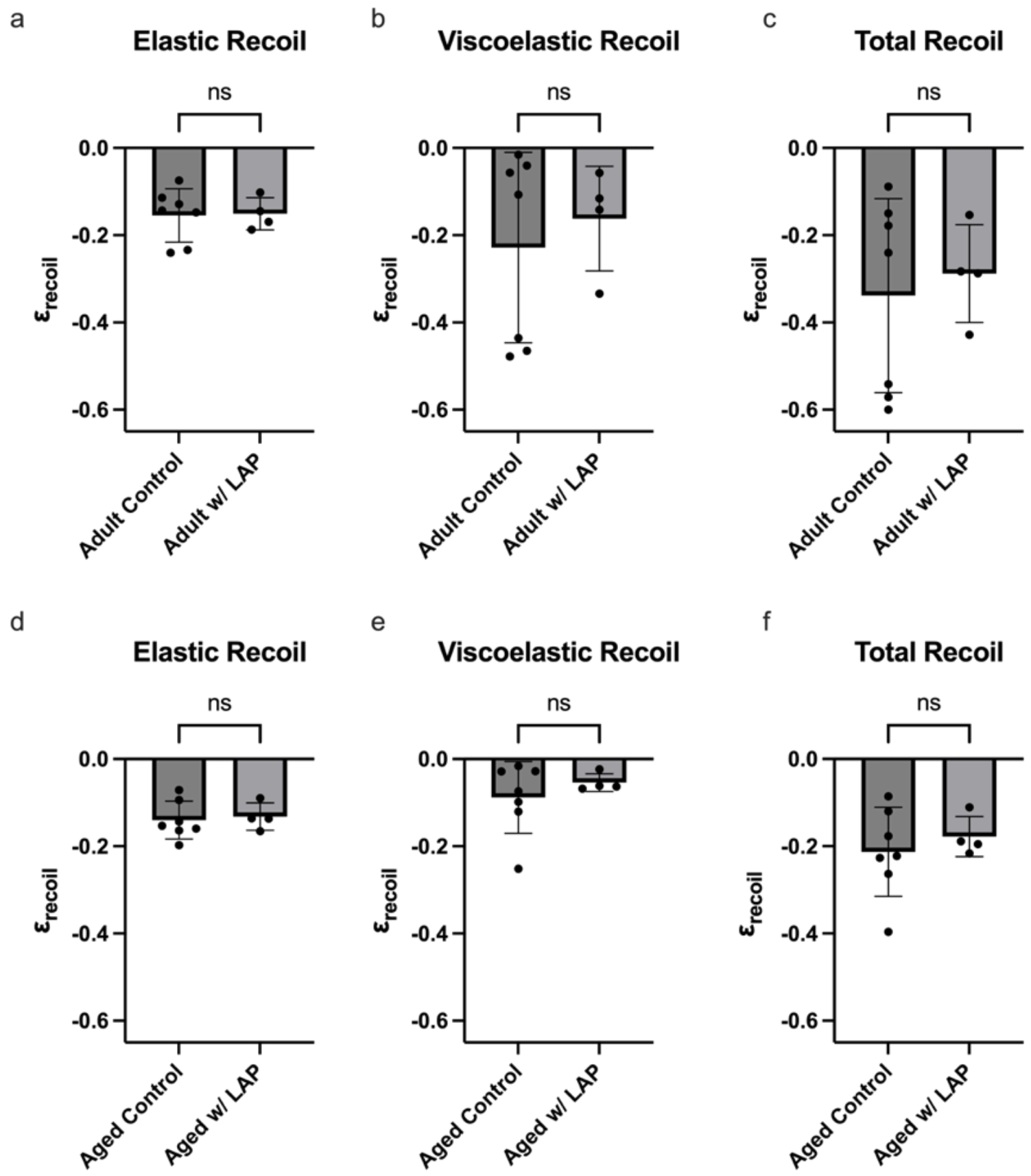

Figure S2. LAP and photocrosslinking did not alter collagen fiber recoil.
a–c) Elastic, viscoelastic, and total collagen fiber recoil following femtosecond laser ablation in adult control tendons and tendons exposed to 0.05 wt/vol% LAP with 3 min of 450 nm light exposure (Adult + LAP). *d–f)* Elastic, viscoelastic, and total collagen fiber recoil following femtosecond laser ablation in aged control tendons and tendons exposed to 0.05 wt/vol% LAP with 3 min of 450 nm light exposure (Aged + LAP). No significant differences were detected between groups (Welch's t-test, $p > 0.05$).

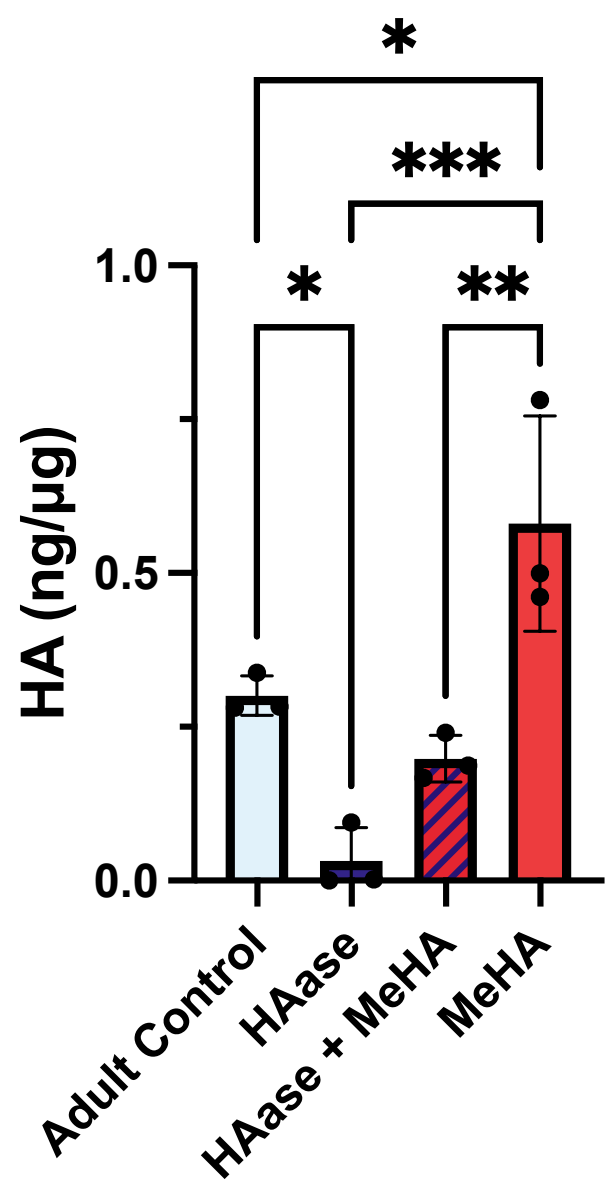

Figure S3: HA content per tendon dry weight measured by ELISA. HAase treatment significantly reduced tendon HA content, while MeHA supplementation restored HA levels. Data are presented as mean ± SD. HA content with treatment was significant (***p<0.001, one-way ANOVA with Dunnett's T3 multiple-comparisons test).

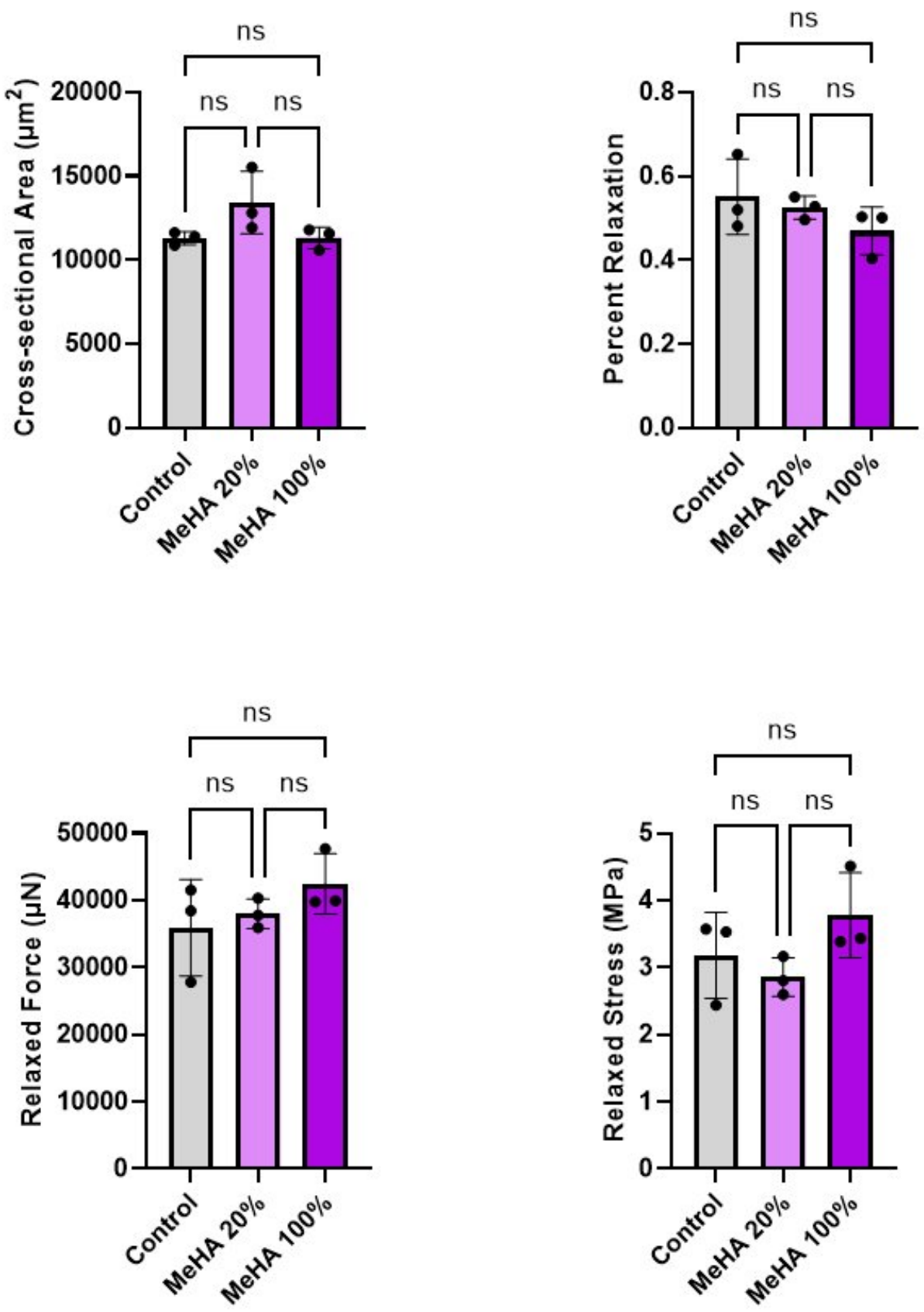

Figure S4. Degree of MeHA methacrylation did not significantly alter macroscale mechanical properties of tendon. Cross-sectional area, percent stress relaxation after 300 s, relaxed force after 300 s, and relaxed stress at the time of ablation. Data represent mean ± SD. Brown–Forsythe one-way ANOVA with Dunnett's T3 post hoc test, n = 3 per group. ns = not significant.

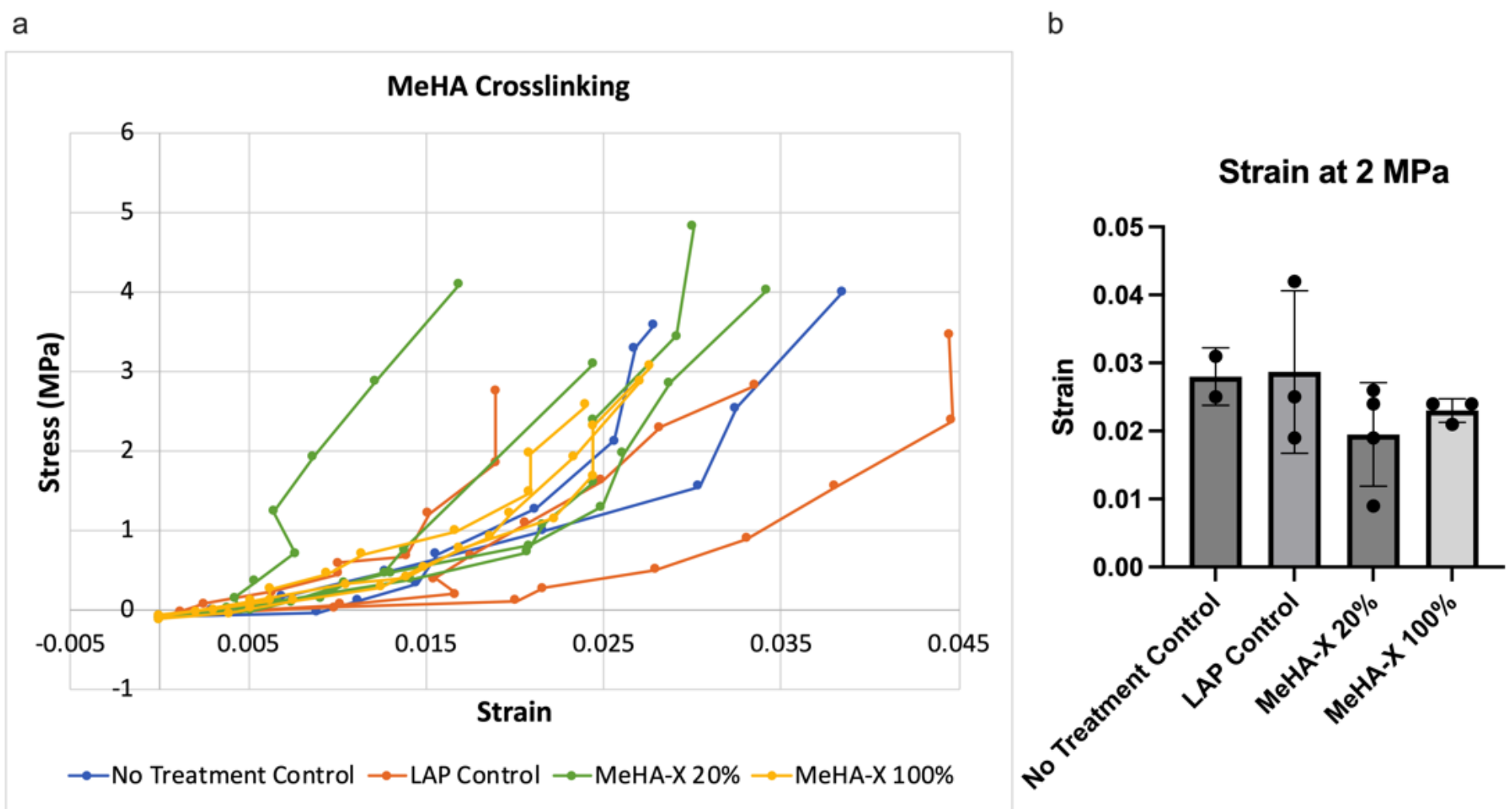

Figure S5. Degree of MeHA methacrylation did not significantly alter tendon tensile behavior. a) Stress–strain curves for control tendons and tendons treated with MeHA-X at varying degrees of methacrylation. b) Strain at 2 MPa stress. Data represent mean ± SD. Brown–Forsythe one-way ANOVA with Dunnett's T3 post hoc test, n = 2-3 per group. No significant differences were detected between groups (ns).

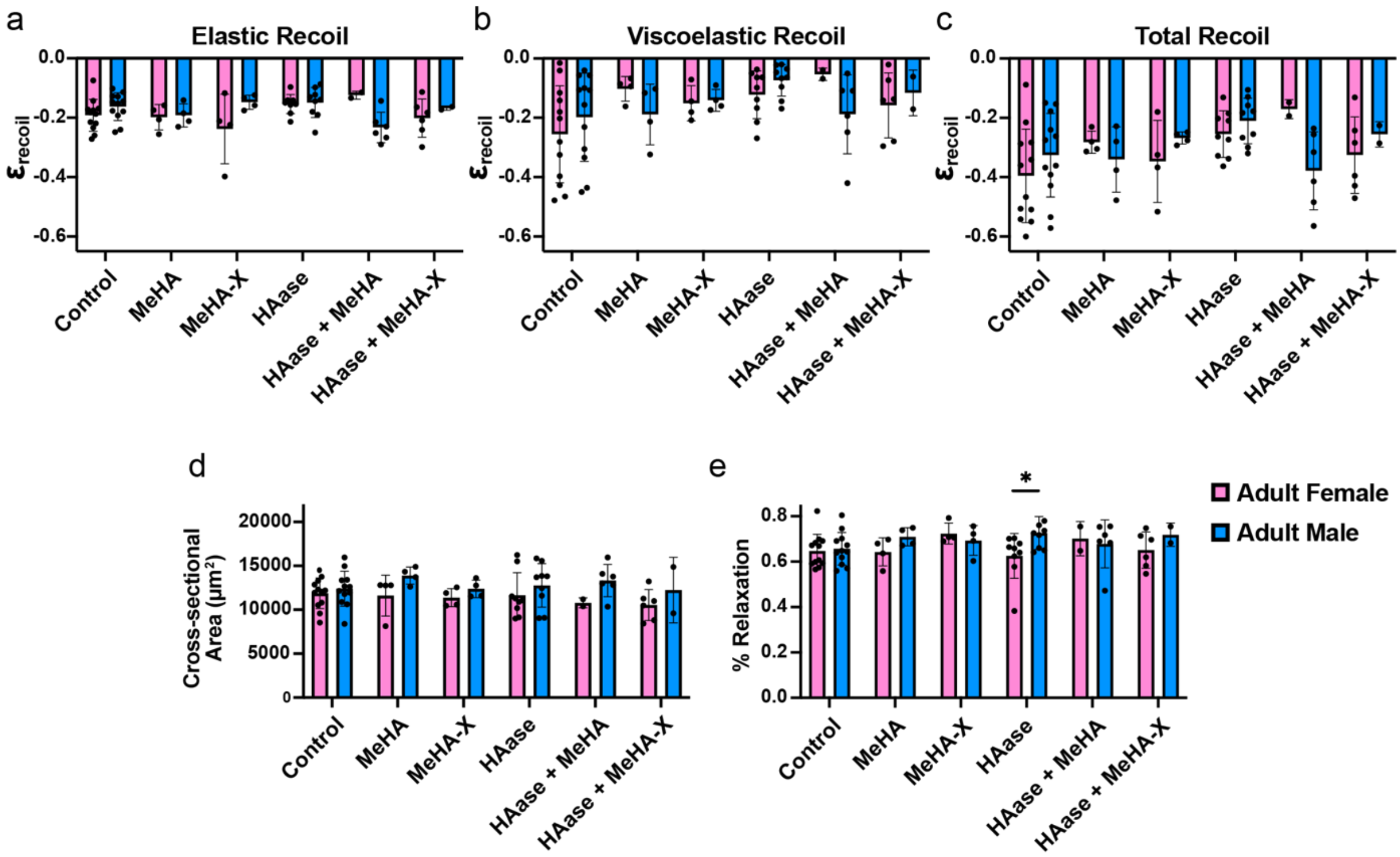

Figure S6. MeHA treatment altered tendon recoil behavior, while tendon cross-sectional area differed by sex. a) Elastic recoil, b) viscoelastic recoil, c) total recoil, d) cross-sectional area, and e) percent stress relaxation for adult female and adult male tendons across treatment groups. Data represent mean ± SD with individual biological replicates shown. Two-way ANOVA revealed a significant sex × treatment interaction for elastic recoil (*$p < 0.05$), significant treatment effects for viscoelastic recoil (*$p < 0.05$) and total recoil (*$p < 0.05$), and a significant effect of sex on cross-sectional area (**$p = 0.007$). n = 6–8 per group.
